# Special Issue on Rate Distortion Theory and Information Theory

**DOI:** 10.3390/e20110825

**Published:** 2018-10-27

**Authors:** Jerry Gibson

**Affiliations:** Department of Electrical and Computer Engineering, University of California, Santa Barbara, CA 93106, USA; gibson@ece.ucsb.edu

**Keywords:** rate distortion bounds, composite source models, conditional rate distortion theory, video codec performance, speech codec performance

Shannon introduced the fields of information theory and rate distortion theory in his landmark 1948 paper [1], where he defined “The Rate for a Source Relative to a Fidelity Evaluation.” Shannon officially coined the term “rate distortion function” in his seminal contribution in 1959 [2]. The 1950s, 1960s and 1970s showed considerable activity on deriving rate distortion functions for various source models and distortion measures and on methods for computing the rate distortion function. Entering the 1980s, attention turned to multiterminal rate distortion problems such as successive refinement of information and multiple descriptions, along with activity pointed toward evaluating rate distortion regions for multiterminal problems. Berger and Gibson [3] provided an overview of rate distortion theory research and its impact on source codec designs for the first 50 years of information theory in 1998. In the subsequent 20 years, research contributions to rate distortion theory have seemingly declined, which is somewhat perplexing, since lossy source compression applications have proliferated in those same decades. Specifically, speech, image, audio and video codecs are ubiquitous in our lives today, with biomedical applications now garnering substantial lossy source compression attention.

As was stated in the invitation to the Special Issue, “It is the goal of this Special Issue to reemphasize the critical accomplishments of rate distortion theory and to highlight new directions in rate distortion theoretic and information theoretic research.” Toward this end, we have collected 10 papers that examine source models that are relevant to real-world sources [4,5,6,7], consider new distortion measures [8], extend prior work in multiterminal source coding [9,10], examine source model identification and coding [11], study rate-distortion optimization for 3D video coding [12] and present a review of the field of information theory and cognition [13]. These papers not only make significant contributions to the rate distortion theory literature, but also highlight new research directions and indicate that there is much more to be done to harness the full promise of rate distortion theory as defined by Shannon.
